# Straight Disclinations in Fractional Nonlocal Medium

**DOI:** 10.3390/ma18081717

**Published:** 2025-04-09

**Authors:** Tamara Kyrylych, Yuriy Povstenko

**Affiliations:** Department of Mathematics and Computer Science, Faculty of Science and Technology, Jan Dlugosz University in Czestochowa, al. Armii Krajowej 13/15, 42-200 Czestochowa, Poland; j.povstenko@ujd.edu.pl

**Keywords:** wedge disclination, twist disclination, nonlocal elasticity, nonlocality kernel, fractional differential equation, Caputo derivative, Mittag–Leffler function

## Abstract

The constitutive equation for a nonlocal stress tensor is represented as an integral with the suitable kernel function. In this paper, the nonlocality kernel is chosen as the Green function of the Cauchy problem for the fractional diffusion equation with the Caputo derivative with respect to the nonlocality parameter. The solutions of nonlocal elasticity problems for the straight wedge and twist disclinations in an infinite medium are obtained in the framework of this new nonlocal theory of elasticity. The Laplace integral transform with respect to the nonlocality parameter is used. It is necessary to emphasize that the transition from the nonlocal to local stress tensor is described by the limiting value of the nonlocality parameter τ→0. The obtained stress fields do not contain nonphysical singularities at the disclination lines.

## 1. Introduction

Real solids contain a large number of different types of defects. In 1907, Volterra [1] considered elasticity problems for a long homogeneous isotropic hollow cylinder. The studied objects were called distortions by Volterra. Later on, defects of the translation type were named dislocations [2], whereas the defects of the rotational type were named disclinations [3]. Translation is described by the Burgers vector b, while rotation is associated with the Frank vector Ω. In 1934, Taylor [4], Orowan [5], and Polanyi [6] showed that the plastic deformation in crystalline solids can be explained in terms of the theory of Volterra dislocations. This insight was critical in developing the modern science of solid mechanics. The literature on dislocations is quite voluminous; we refer to the well-known books [7,8,9,10,11,12,13], where additional references can be found. For a long time, disclinations were in the shadow of dislocations, but this situation was changed after the appearance of a series of papers by deWit [14,15] (see, for example, [16,17,18,19,20,21,22,23], and the extensive bibliography in [24,25,26,27,28]). There are strict relations between dislocations and disclinations [15,29,30]. Dislocations can end at disclinations. Disclinations can be modelled by groups of dislocations with some density. In this case, the stress fields of disclinations can be obtained as the corresponding integrals of the stress fields caused by dislocation arrays. However, identifying which description is more appropriate in a specific case is an issue [26].

The problem of modeling stress and strain fields around dislocations and disclinations has attracted much attention from researchers. Effective methods for classical elasticity cannot describe the situation in the immediate vicinity of an imperfection, as nonphysical singularities appear. Therefore, regular attempts have been made to improve the classical elastic models of crystal defects, for instance, combining the elastic and discrete approaches for a better description of the highly distorted region near a defect. In the Frenkel–Kontorova model [31], the dislocation is considered as a set of particles coupled by nearest-nieghbour interaction and moving in a periodic potential. The modern state of studies based on the Frenkel–Kontorova model is presented in [32,33]. Another model that takes into account the discrete structure of the crystal and describes the core of the dislocation is the Peierls–Nabarro model [34,35]. The assumption of this model is that the dislocation is characterized by the elastic energy due to a finite density of dislocations and the misfit energy, which results from the nonlinear atomic interaction in the glide plane. The semi-discrete approach, according to which the crystal with dislocation is divided into two parts (a discrete lattice and an elastic continuum), has been discussed extensively in [36,37,38,39].

Great advances in improving classical elasticity solutions for dislocations and disclinations have been made by the application of different generalized theories. Straight dislocations [40,41,42,43,44,45] and disclinations [46] were studied in different theories of nonlocal elasticity, as well as in the framework of the comparable strain gradient elasticity [47,48,49,50,51,52]. The interested reader is also referred to the approach developed in the papers [53,54,55].

In the present paper, the solutions of nonlocal elasticity problems for the straight wedge and twist disclinations in an infinite solid are obtained in the framework of the new nonlocal theory of elasticity, in which the nonlocality kernel is chosen as the Green function of the Cauchy problem for the fractional diffusion operator with the Caputo derivative with respect to the nonlocality parameter. In the literature, other kernels were also considered as the Green functions for some differential operators [56,57,58,59].

To solve these problems, the components of the Laplacian of the symmetric second-order tensor in cylindrical coordinates are needed. Such components were obtained in the paper [46]. A coupled systems of equations are split, introducing new unknown functions. The Laplace transform with respect to the nonlocality parameter is used. The immediate application of the Hankel transform is impossible due to the logarithmic term appearing in the classical elasticity solution. For this reason, a special analysis of the modified Bessel functions of the peculiar argument has been carried out.

The paper is organized as follows. The basic equations of the fractional nonlocal elasticity are presented in Section 2. The solution for the straight wedge disclination is obtained in Section 3. Section 4 is dedicated to the solution for the straight twist disclination. Finally, concluding remarks are presented in Section 5.

## 2. Fractional Nonlocal Elasticity

According to the theory of nonlocal elasticity, the stress tensor at a reference point of a solid depends not only on the strains at this point but also on strains at all other points of the solid. For example, for a linear isotropic nonlocal elastic body

(1) $$t\left(x\right)=\int_{V}K\left(|x-x^{\prime }|\right)\sigma \left(x^{\prime }\right)dv\left(x^{\prime }\right),$$

(2) $$\sigma \left(x^{\prime }\right)=2\mu \;e\left(x^{\prime }\right)+\lambda \;\mathrm{tr}\;e\left(x^{\prime }\right)I,$$

where $\sigma \left(x^{\prime }\right)$ and $t\left(x\right)$ are the classical and nonlocal stress tensors, $x^{\prime }$ and x are the running and reference points, e is the linear strain tensor, λ and μ denote the Lamé constants, and I stands for the unit tensor.

The properties of the nonlocal kernel $K\left(x\right)$ were investigated by Eringen [43,44]. In particular, Eringen considered the nonlocal kernel as the Green function of the Cauchy problem for the diffusion operator:

(3) $$\frac{\partial K\left(x,\tau \right)}{\partial \tau }=\Delta \;K\left(x,\tau \right),$$

(4) $$\tau =0:\;\;\;K\left(x,\tau \right)=\delta \left(x\right),$$

which results in the kernel

(5) $$K\left(x,\tau \right)=\frac{1}{{\left(2\sqrt{\pi \tau }\right)}^{n}}\;\exp\left(-\frac{{\left|x\right|}^{2}}{4\tau }\right),$$

with n=1,2,3 correlated with a number of spatial variables.

This approach results in the Cauchy problem for the nonlocal stress tensor

(6) $$\frac{\partial t\left(x,\tau \right)}{\partial \tau }=\Delta \;t\left(x,\tau \right),$$

(7) $$\tau =0:\;\;\;t\left(x,\tau \right)=\sigma \left(x\right).$$

Fractional calculus has many applications in different areas of science and was also used in the formulation of nonlocal elasticity approaches (see, for example, [60,61,62,63,64,65]). Fractional nonlocal elasticity theory with the nonlocality kernel being the Green function of the Cauchy problem for fractional diffusion equation with the Caputo derivative with respect to nonlocality parameter $\partial^{\alpha }/\partial \tau^{\alpha }$ and fractional Laplace operator (Riesz operator) ${\left(-\Delta \right)}^{\beta /2}$ was proposed in [66]. In the present paper, we restrict our attention to the case β=2. In the proposed theory [66], the nonlocal modulus satisfies the equation

(8) $$\frac{\partial^{\alpha }K\left(x,\tau \right)}{\partial \tau^{\alpha }}=a\;\Delta \;K\left(x,\tau \right),\;0<\alpha \leq 1,$$

(9) $$\tau =0:\;\;\;K\left(x,\tau \right)=\delta \left(x\right).$$

The Caputo fractional derivative [67,68] is defined as

(10) $$\frac{d^{\alpha }f\left(\tau \right)}{d\tau^{\alpha }}=\frac{1}{\Gamma (m-\alpha )}\int_{0}^{\tau }{\left(\tau -u\right)}^{m-\alpha -1}\;\frac{d^{m}f\left(u\right)}{du^{m}}\;du,\;m-1<\alpha <m.$$

Here, Γ(α) is the gamma function. The Caputo derivative (10) has the Laplace transform rule:

(11) $$L\left\{\frac{d^{\alpha }f\left(\tau \right)}{d\tau^{\alpha }}\right\}=s^{\alpha }f^{*}\left(s\right)-\sum_{k=0}^{m-1}f^{\left(k\right)}\left(0^{+}\right)s^{\alpha -1-k},\;m-1<\alpha <m,$$

where the asterisk denotes the Laplace transform, with *s* being the transform variable.

Instead of the solution (5), the nonlocal kernel $K\left(x,\tau \right)$ for a different number *n* of spatial variables is expressed in terms of the Mittag–Leffler function, Mainardi function, and Wright function (see [66]).

The Cauchy problem for the nonlocality kernel $K\left(x,\tau \right)$ (8) and (9) results in the Cauchy problem for the nonlocal stress tensor

(12) $$\frac{\partial^{\alpha }t\left(x,\tau \right)}{\partial \tau^{\alpha }}=a\;\Delta \;t\left(x,\tau \right),\;0<\alpha \leq 1,$$

(13) $$\tau =0:\;\;\;t\left(x,\tau \right)=\sigma \left(x\right).$$

It is necessary to accentuate that in Equations (3), (6), (8) and (12), the letter τ does not denote time; τ is the nonlocality parameter. The conditions (7) and (13) do not correspond to the initial time value, but describe the transition from the nonlocal to local stress tensor. Eringen in his publications [43,44] showed how the nonlocality parameter τ can be connected with atomic lattice theory. The parameter τ is associated with a characteristic length ratio l/L, where *l* is an internal characteristic length (for example, the lattice parameter) and *L* is an external characteristic length (wavelength, solid size, etc.). Eringen [43,44] and Kunin [69,70] provided a detailed analysis of relations between nonlocal elasticity theory and Debye quasi-continuum and the Born-Kármán model of atomic lattice dynamics and atomic dispersion curves. Similar considerations can be applied to the proposed theory. We also note that Eringen [42,44] considered Equations (3) and (6) without the coefficient *a*; we have written the coefficient *a* in Equations (8) and (12) to have the possibility of studying different limiting cases of the solutions.

## 3. Straight Wedge Disclination

**Figure 1:**
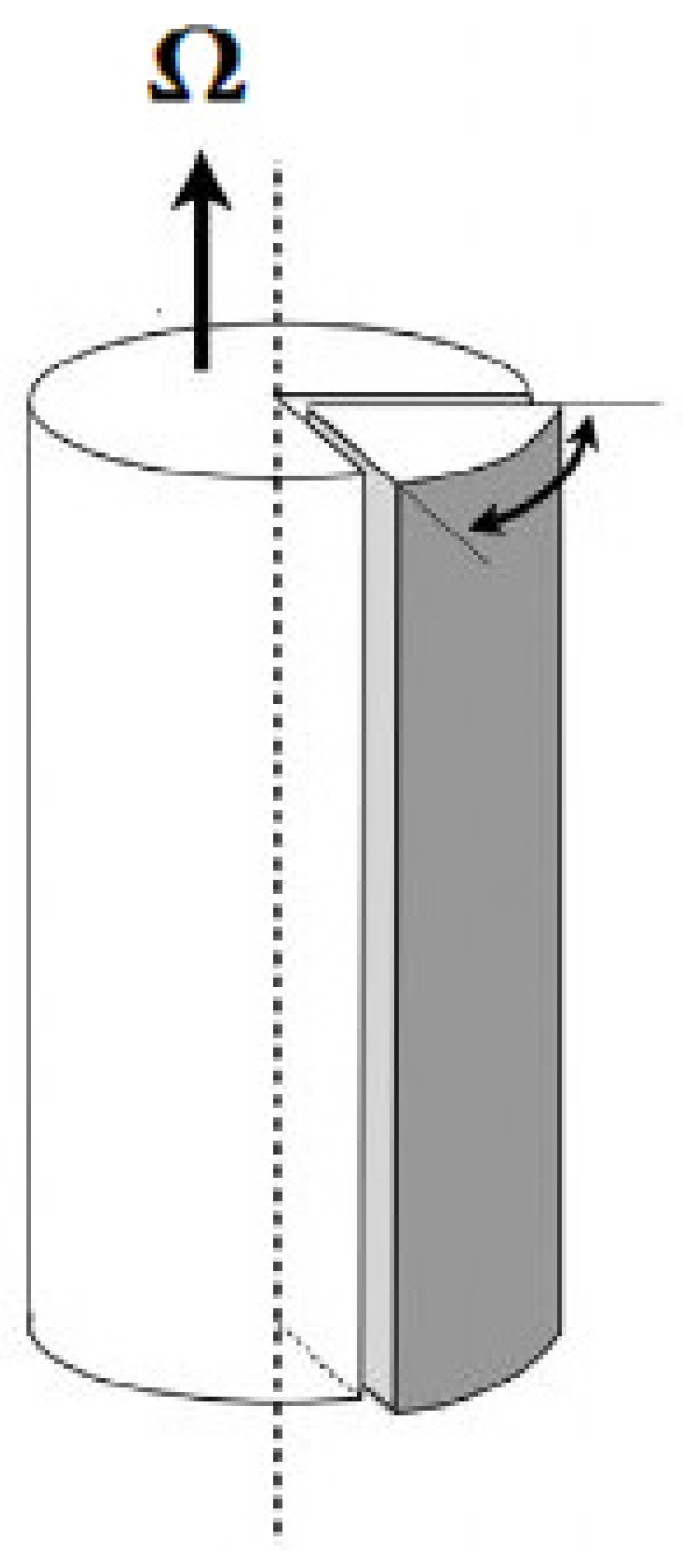
Schematic representation of a straight wedge disclination with the Frank vector $\Omega_{3}$.

Assume the *z*-axis as the disclination line and consider the straight wedge disclination with the Frank vector $\Omega =\left(0,0,\Omega_{3}\right)$. A drawing of the wedge disclination is presented in Figure 1.

Under the conditions of the plane strain, the classical elasticity stress tensor for the straight wedge disclination has the following components [15] in cylindrical coordinates:

(14) $$\begin{array}{ccc} \sigma_{rr} & = & A\left[\ln\left(r/l\right)+\frac{\nu }{1-2\nu }\right], \end{array}$$

(15) $$\begin{array}{ccc} \sigma_{\theta \theta } & = & A\left[\ln\left(r/l\right)+\frac{1-\nu }{1-2\nu }\right], \end{array}$$

(16) $$\begin{array}{ccc} \sigma_{zz} & = & A\left[2\nu \ln\left(r/l\right)+\frac{\nu }{1-2\nu }\right], \end{array}$$

(17) $$\begin{array}{ccc} \sigma_{r\theta } & = & 0,\;\sigma_{rz}=0,\;\sigma_{\theta z}=0, \end{array}$$

where

(18) $$A=\frac{\mu \Omega_{3}}{2\pi (1-\nu )},$$

where ν is the Poisson ratio.

It should be noted that deWit [15] wrote the corresponding equations with the term lnr. We have slightly changed Equations (14)–(16) in comparison with the original equations of deWit, introducing the nondimensional quantity r/l under the logarithm symbol. The specific expression for the characteristic length *l* will be discussed below.

From Equations (12) and (13), using the components of the Laplacian of the stress tensor presented in Appendix A and the components of the local stress tensor (14)–(17), we obtain

(19) $$\begin{array}{ccc} & & \frac{\partial^{\alpha }t_{rr}}{\partial \tau^{\alpha }}=a\left[\frac{\partial^{2}t_{rr}}{\partial r^{2}}+\frac{1}{r}\;\frac{\partial t_{rr}}{\partial r}-\frac{2}{r^{2}}\;\left(t_{rr}-t_{\theta \theta }\right)\right], \end{array}$$

(20) $$\begin{array}{ccc} & & \tau =0:\;t_{rr}=A\left[\ln\left(r/l\right)+\frac{\nu }{1-2\nu }\right]; \end{array}$$

(21) $$\begin{array}{ccc} & & \frac{\partial^{\alpha }t_{\theta \theta }}{\partial \tau^{\alpha }}=a\left[\frac{\partial^{2}t_{\theta \theta }}{\partial r^{2}}+\frac{1}{r}\;\frac{\partial t_{\theta \theta }}{\partial r}+\frac{2}{r^{2}}\;\left(t_{rr}-t_{\theta \theta }\right)\right], \end{array}$$

(22) $$\begin{array}{ccc} & & \tau =0:\;t_{\theta \theta }=A\left[\ln\left(r/l\right)+\frac{1-\nu }{1-2\nu }\right]; \end{array}$$

(23) $$\begin{array}{ccc} & & \frac{\partial^{\alpha }t_{zz}}{\partial \tau^{\alpha }}=a\left[\frac{\partial^{2}t_{zz}}{\partial r^{2}}+\frac{1}{r}\;\frac{\partial t_{zz}}{\partial r}\right], \end{array}$$

(24) $$\begin{array}{ccc} & & \tau =0:\;t_{zz}=A\left[2\nu \ln\left(r/l\right)+\frac{\nu }{1-2\nu }\right]. \end{array}$$

Now, we introduce two auxiliary functions

(25) $$\begin{array}{ccc} f(r,\tau ) & = & t_{rr}\left(r,\tau \right)+t_{\theta \theta }\left(r,\tau \right), \end{array}$$

(26) $$\begin{array}{ccc} g(r,\tau ) & = & t_{rr}\left(r,\tau \right)-t_{\theta \theta }\left(r,\tau \right) \end{array}$$

which allow us to split Equations (19)–(22):

(27) $$\begin{array}{ccc} & & \frac{\partial^{\alpha }f}{\partial \tau^{\alpha }}=a\left(\frac{\partial^{2}f}{\partial r^{2}}+\frac{1}{r}\;\frac{\partial f}{\partial r}\right), \end{array}$$

(28) $$\begin{array}{ccc} & & \tau =0:\;f=A\left[2\ln\left(r/l\right)+\frac{1}{1-2\nu }\right], \end{array}$$

(29) $$\begin{array}{ccc} & & \frac{\partial^{\alpha }g}{\partial \tau^{\alpha }}=a\left(\frac{\partial^{2}g}{\partial r^{2}}+\frac{1}{r}\;\frac{\partial g}{\partial r}-\frac{4}{r^{2}}\;g\right), \end{array}$$

(30) $$\begin{array}{ccc} & & \tau =0:\;g=-A. \end{array}$$

In this case,

(31) $$\begin{array}{ccc} t_{rr}\left(r,\tau \right) & = & \frac{1}{2}\left[f(r,\tau )+g(r,\tau )\right], \end{array}$$

(32) $$\begin{array}{ccc} t_{\theta \theta }\left(r,\tau \right) & = & \frac{1}{2}\left[f(r,\tau )-g(r,\tau )\right], \end{array}$$

(33) $$\begin{array}{ccc} t_{zz}\left(r,\tau \right) & = & \nu \;f(r,\tau ). \end{array}$$

The Laplace transform with respect to the nonlocality parameter τ gives

(34) $$\begin{array}{ccc} & & \frac{\partial^{2}f^{*}}{\partial r^{2}}+\frac{1}{r}\;\frac{\partial f^{*}}{\partial r}-\frac{s^{\alpha }}{a}\;f^{*}=-\frac{s^{\alpha -1}}{a}A\left[2\ln\left(r/l\right)+\frac{1}{1-2\nu }\right], \end{array}$$

(35) $$\begin{array}{ccc} & & \frac{\partial^{2}g^{*}}{\partial r^{2}}+\frac{1}{r}\;\frac{\partial g^{*}}{\partial r}-\frac{4}{r^{2}}\;g^{*}-\frac{s^{\alpha }}{a}\;g^{*}=\frac{s^{\alpha -1}}{a}A. \end{array}$$

The solutions of equations of such a type are expressed in terms of the modified Bessel functions of the zeroth and second order

(36) $$I_{0}\left(\sqrt{\frac{s^{\alpha }}{a}}\;r\right),\;K_{0}\left(\sqrt{\frac{s^{\alpha }}{a}}\;r\right),\;I_{2}\left(\sqrt{\frac{s^{\alpha }}{a}}\;r\right),\;K_{2}\left(\sqrt{\frac{s^{\alpha }}{a}}\;r\right).$$

The modified Bessel functions $I_{0}\left(r\right)$ and $I_{2}\left(r\right)$ have exponential divergence at infinity, and the modified Bessel functions $K_{0}\left(r\right)$ and $K_{2}\left(r\right)$ have the following expansion at the origin [71]:

(37) $$K_{0}\left(r\right)∼-\ln r,\;K_{2}\left(r\right)∼\frac{2}{r^{2}}-\frac{1}{2}.$$

The regularity requirement of the solution at the origin allows us to interprete the constant *l* as $l=\sqrt{a/s^{\alpha }}$ and obtain the solution of Equations (34) and (35) as:

(38) $$\begin{array}{ccc} f^{*}\left(r,s\right) & = & \frac{2A}{s}\;K_{0}\left(\sqrt{\frac{s^{\alpha }}{a}}\;r\right)+\frac{A}{s}\left[2\ln\left(\sqrt{\frac{s^{\alpha }}{a}}\;r\right)+\frac{1}{1-2\nu }\right], \end{array}$$

(39) $$\begin{array}{ccc} g^{*}\left(r,s\right) & = & -\frac{2A}{s}\;K_{2}\left(\sqrt{\frac{s^{\alpha }}{a}}\;r\right)-\frac{A}{s}+\frac{4aA}{s^{1+\alpha }r^{2}}. \end{array}$$

There are no explicit formulae for the inverse Laplace transforms

(40) $$L^{-1}\left\{\frac{1}{s}\;K_{0}\left(\sqrt{\frac{s^{\alpha }}{a}}\;r\right)\right\}\;\mathrm{and}\;L^{-1}\left\{\frac{1}{s}\;K_{2}\left(\sqrt{\frac{s^{\alpha }}{a}}\;r\right)\right\}$$

for arbitrary α. For this reason, we introduce the functions

(41) $$f_{\left(0\right)}^{*}\left(r,s\right)=\frac{2A}{s}\;K_{0}\left(\sqrt{\frac{s^{\alpha }}{a}}\;r\right)\;\mathrm{and}\;g_{\left(2\right)}^{*}\left(r,s\right)=-\frac{2A}{s}\;K_{2}\left(\sqrt{\frac{s^{\alpha }}{a}}\;r\right)$$

and treat them in the following way.

Using Equation (A8) from Appendix B, we evaluate the Hankel transform of the zeroth order of the function $f_{\left(0\right)}^{*}\left(r,s\right)$,

(42) $${\hat{f}}_{\left(0\right)}^{*}\left(\xi ,s\right)=\frac{2aA}{s\left(s^{\alpha }+a\xi^{2}\right)}=\frac{2A}{\xi^{2}}\left(\frac{1}{s}-\frac{s^{\alpha -1}}{s^{\alpha }+a\xi^{2}}\right),$$

where the hat denotes the Hankel transform with respect to the radial coordinate *r*, and ξ is the transform variable.

The inverse Laplace transforms [72]

(43) $$L^{-1}\left\{\frac{\ln s}{s}\right\}=-\ln\tau -\gamma ,$$

where γ=0.57721566… is the Euler constant, and [67,68]

(44) $$L^{-1}\left\{\frac{s^{\alpha -1}}{s^{\alpha }+a\xi^{2}}\right\}=E_{\alpha }\left(-a\xi^{2}\tau^{\alpha }\right),$$

where $E_{\alpha }\left(z\right)$ is the Mittag–Leffler function

(45) $$E_{\alpha }\left(z\right)=\sum_{k=0}^{\infty }\frac{z^{k}}{\Gamma (\alpha k+1)},\;\;\;\alpha >0,\;\;z\in C,$$

and a subsequent inverse Hankel transform allows us to obtain the expression for the function f(r,τ),

(46) $$\begin{array}{ccc} f(r,\tau ) & = & A\left[2\ln\left(\frac{r}{\sqrt{a}\tau^{\alpha /2}}\right)+\frac{1}{1-2\nu }-\alpha \gamma \right]\\ & + & 2A\int_{0}^{\infty }\frac{1-E_{\alpha }\left(-a\xi^{2}\tau^{\alpha }\right)}{\xi }\;J_{0}\left(r\xi \right)\;d\xi . \end{array}$$

Similar treatment is carried out for the function $g_{\left(2\right)}^{*}\left(r,s\right)$. Applying the Hankel transform of the second order to $g_{\left(2\right)}^{*}\left(r,s\right)$ and taking into account integral (A9) from Appendix B, after some mathematical transformations, we arrive at the expression

(47) $$\begin{array}{ccc} g(r,\tau ) & = & -A-2A\int_{0}^{\infty }\frac{1-E_{\alpha }\left(-a\xi^{2}\tau^{\alpha }\right)}{\xi }\;J_{0}\left(r\xi \right)d\xi \\ & + & \frac{4A}{r}\;\int_{0}^{\infty }\frac{1-E_{\alpha }\left(-a\xi^{2}\tau^{\alpha }\right)}{\xi^{2}}\;J_{1}\left(r\xi \right)d\xi \end{array}$$

or, using the recurrence equation for the function $J_{2}\left(r\right)$ in terms of $J_{0}\left(r\right)$ and $J_{1}\left(r\right)$,

(48) $$g\left(r,\tau \right)=-2A\int_{0}^{\infty }\frac{E_{\alpha }\left(-a\xi^{2}\tau^{\alpha }\right)}{\xi }\;J_{2}\left(r\xi \right)\;d\xi .$$

Equation (48) can also be obtained applying the Hankel transform of the second order immediately to Equation (35), taking into account integral (A10) from Appendix B. This confirms the interpretation of the characteristic length as $l=\sqrt{a/s^{\alpha }}$ and the approach based on introducing the functions $f_{\left(0\right)}^{*}\left(r,s\right)$ and $g_{\left(2\right)}^{*}\left(r,s\right)$ by Equation (41). It should be emphasized that application of the Hankel transform immediately to Equation (38) is impossible due to the logarithmic term in the right-hand side of this equation.

Finally, the components of the nonlocal stress tensor take the form

(49) $$\begin{array}{ccc} t_{rr}\left(r,\tau \right) & = & \frac{A}{2}\left[2\ln\left(\frac{r}{\sqrt{a}\tau^{\alpha /2}}\right)+\frac{1}{1-2\nu }-\alpha \gamma \right]\\ & + & A\int_{0}^{\infty }\frac{1-E_{\alpha }\left(-a\xi^{2}\tau^{\alpha }\right)}{\xi }\;J_{0}\left(r\xi \right)d\xi \\ & - & A\int_{0}^{\infty }\frac{E_{\alpha }\left(-a\xi^{2}\tau^{\alpha }\right)}{\xi }\;J_{2}\left(r\xi \right)d\xi , \end{array}$$

(50) $$\begin{array}{ccc} t_{\theta \theta }\left(r,\tau \right) & = & \frac{A}{2}\left[2\ln\left(\frac{r}{\sqrt{a}\tau^{\alpha /2}}\right)+\frac{1}{1-2\nu }-\alpha \gamma \right]\\ & + & A\int_{0}^{\infty }\frac{1-E_{\alpha }\left(-a\xi^{2}\tau^{\alpha }\right)}{\xi }\;J_{0}\left(r\xi \right)d\xi \\ & + & A\int_{0}^{\infty }\frac{E_{\alpha }\left(-a\xi^{2}\tau^{\alpha }\right)}{\xi }\;J_{2}\left(r\xi \right)d\xi , \end{array}$$

or, accounting for the recurrence equation for the Bessel function,

(51) $$\begin{array}{ccc} t_{rr}\left(r,\tau \right) & = & A\left[\ln\left(\frac{r}{\sqrt{a}\tau^{\alpha /2}}\right)+\frac{\nu }{1-2\nu }-\frac{1}{2}\;\alpha \gamma \right]\\ & + & \frac{2A}{r}\;\int_{0}^{\infty }\frac{1-E_{\alpha }\left(-a\xi^{2}\tau^{\alpha }\right)}{\xi^{2}}\;J_{1}\left(r\xi \right)d\xi , \end{array}$$

(52) $$\begin{array}{ccc} t_{\theta \theta }\left(r,\tau \right) & = & A\left[\ln\left(\frac{r}{\sqrt{a}\tau^{\alpha /2}}\right)+\frac{1-\nu }{1-2\nu }-\frac{1}{2}\;\alpha \gamma \right]\\ & + & 2A\int_{0}^{\infty }\frac{1-E_{\alpha }\left(-a\xi^{2}\tau^{\alpha }\right)}{\xi }\;J_{0}\left(r\xi \right)d\xi \\ & - & \frac{2A}{r}\;\int_{0}^{\infty }\frac{1-E_{\alpha }\left(-a\xi^{2}\tau^{\alpha }\right)}{\xi^{2}}\;J_{1}\left(r\xi \right)d\xi . \end{array}$$

Consider particular cases of the obtained solution. If α=1, then $E_{1}\left(-a\xi^{2}\tau \right)=\exp\left(-a\xi^{2}\tau \right)$, and using integrals (A11)–(A13) from Appendix B, we arrive at

(53) $$\begin{array}{ccc} t_{rr}\left(r,\tau \right) & = & A\left[\ln\left(\frac{r}{\sqrt{a\tau }}\right)+\frac{\nu }{1-2\nu }-\frac{1}{2}\;\gamma \right]+\frac{2Aa\tau }{r^{2}}\left[1-E_{2}\left(\frac{r^{2}}{4a\tau }\right)\right], \end{array}$$

(54) $$\begin{array}{ccc} t_{\theta \theta }\left(r,\tau \right) & = & A\left[\ln\left(\frac{r}{\sqrt{a\tau }}\right)+\frac{1-\nu }{1-2\nu }-\frac{1}{2}\;\gamma \right]\\ & - & \frac{2Aa\tau }{r^{2}}\left[1-\exp\left(-\frac{r^{2}}{4a\tau }\right)\right]+\frac{A}{2}\;E_{1}\left(\frac{r^{2}}{4a\tau }\right). \end{array}$$

Another particular case corresponds to α→0 with $E_{0}\left(-a\xi^{2}\right)=1/\left(1+a\xi^{2}\right)$, which results in

(55) $$t_{rr}\left(r,\tau \right)=A\;\left[\ln\left(\frac{r}{\sqrt{a}}\right)+\frac{\nu }{1-2\nu }+\frac{2a}{r^{2}}-\frac{2\sqrt{a}}{r}\;K_{1}\left(\frac{r}{\sqrt{a}}\right)\right],$$

(56) $$t_{\theta \theta }\left(r,\tau \right)=A\;\left[\ln\left(\frac{r}{\sqrt{a}}\right)+\frac{1-\nu }{1-2\nu }-\frac{2a}{r^{2}}+K_{0}\left(\frac{r}{\sqrt{a}}\right)+K_{2}\left(\frac{r}{\sqrt{a}}\right)\right].$$

To obtain the solution (55), (56), we use integrals (A15) and (A16) from Appendix B and the recurrence equations for the modified Bessel functions $K_{n}\left(r\right)$. Both particular cases α=1 and α→0 were earlier studied in [46].

**Figure 2:**
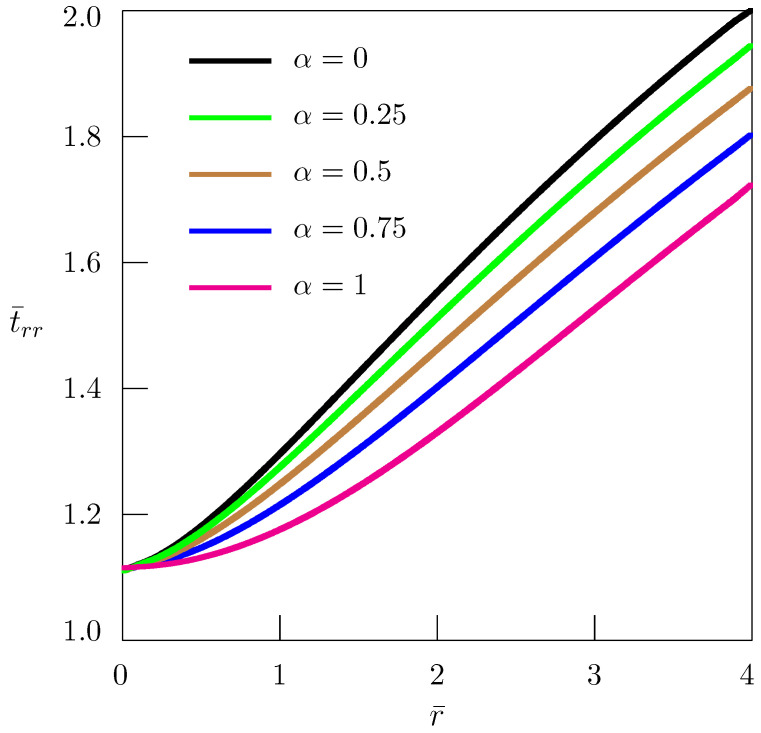
Dependence of stress ${\bar{t}}_{rr}$ on distance for straight wedge disclination.

Figure 2 shows the dependence of nondimensional stress on nondimensional distance (similarity variable) with

(57) $${\bar{t}}_{rr}=\frac{t_{rr}}{A},\;\bar{r}=\frac{r}{\sqrt{a}\tau^{\alpha /2}}.$$

In numerical simulations, we assume ν=0.25. It follows from Figure 2 that at the disclination line ${\bar{t}}_{rr}\left(0\right)\approx 1.1$. Hence, from Equations (18) and (57), for example, taking $\Omega_{3}=\pi /30$, we obtain $t_{rr}\left(0\right)\approx 0.024\;\mu$. This approximate estimate is quite acceptable.

## 4. Straight Twist Disclination

**Figure 3:**
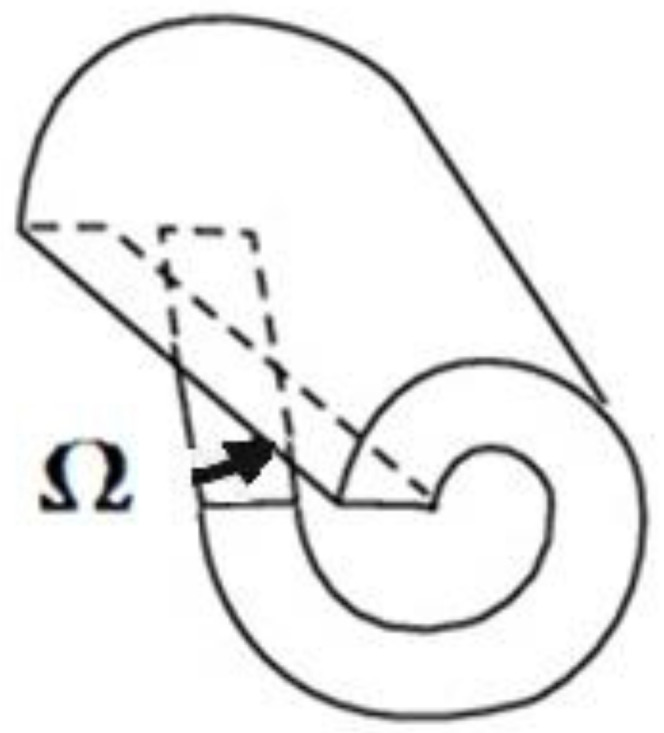
Schematic representation of a straight twist disclination with the Frank vector $\Omega_{2}$.

A drawing of the straight twist disclination with Frank vector $\Omega =\left(0,\Omega_{2},0\right)$ is shown in Figure 3.

The classical elasticity stress tensor for such a twist disclination has the following components [15] in cylindrical coordinates $\left(r,\theta ,z\right)$:

(58) $$\begin{array}{ccc} \sigma_{rr} & = & -B\;\frac{z}{r}\;\sin\theta , \end{array}$$

(59) $$\begin{array}{ccc} \sigma_{\theta \theta } & = & -B\;\frac{z}{r}\;\sin\theta , \end{array}$$

(60) $$\begin{array}{ccc} \sigma_{zz} & = & -2\nu \;B\;\frac{z}{r}\;\sin\theta \end{array}$$

(61) σrθ=−Bzrcosθ,

(62) $$\begin{array}{ccc} \sigma_{rz} & = & -B\left(1-2\nu \right)\ln\left(r/l\right)\sin\theta , \end{array}$$

(63) $$\begin{array}{ccc} \sigma_{\theta z} & = & -B\left[\left(1-2\nu \right)\ln\left(r/l\right)+1\right]\cos\theta , \end{array}$$

where

(64) $$B=\frac{\mu \;\Omega_{2}}{2\pi (1-\nu )}.$$

We assume that the components of the nonlocal stress tensor are represented as

(65) $$\begin{array}{ccc} t_{rr}\left(r,\theta ,z,\tau \right) & = & T_{rr}\left(r,z,\tau \right)\;\sin\theta , \end{array}$$

(66) $$\begin{array}{ccc} t_{\theta \theta }\left(r,\theta ,z,\tau \right) & = & T_{\theta \theta }\left(r,z,\tau \right)\;\sin\theta , \end{array}$$

(67) $$\begin{array}{ccc} t_{zz}\left(r,\theta ,z,\tau \right) & = & T_{zz}\left(r,z,\tau \right)\;\sin\theta , \end{array}$$

(68) $$\begin{array}{ccc} t_{r\theta }\left(r,\theta ,z,\tau \right) & = & T_{r\theta }\left(r,z,\tau \right)\;\cos\theta , \end{array}$$

(69) $$\begin{array}{ccc} t_{rz}\left(r,\theta ,\tau \right) & = & T_{rz}\left(r,\tau \right)\;\sin\theta , \end{array}$$

(70) $$\begin{array}{ccc} t_{\theta z}\left(r,\theta ,\tau \right) & = & T_{\theta z}\left(r,\tau \right)\;\cos\theta . \end{array}$$

Using the components of the Laplacian of the stress tensor (see Appendix A), from Equations (12), (13), (58)–(63) and (65)–(70), we obtain a coupled system of equations for determining the unknown coefficients:

(71) $$\begin{array}{ccc} & & \frac{\partial^{\alpha }T_{rr}}{\partial \tau^{\alpha }}=a\left[\frac{\partial^{2}T_{rr}}{\partial r^{2}}+\frac{1}{r}\;\frac{\partial T_{rr}}{\partial r}-\frac{1}{r^{2}}\;T_{rr}+\frac{4}{r^{2}}\;T_{r\theta }-\frac{2}{r^{2}}\;\left(T_{rr}-T_{\theta \theta }\right)\right], \end{array}$$

(72) $$\begin{array}{ccc} & & \tau =0:\;T_{rr}=-B\;\frac{z}{r}; \end{array}$$

(73) $$\begin{array}{ccc} & & \frac{\partial^{\alpha }T_{\theta \theta }}{\partial \tau^{\alpha }}=a\left[\frac{\partial^{2}T_{\theta \theta }}{\partial r^{2}}+\frac{1}{r}\;\frac{\partial T_{\theta \theta }}{\partial r}-\frac{1}{r^{2}}\;T_{\theta \theta }-\frac{4}{r^{2}}\;T_{r\theta }+\frac{2}{r^{2}}\;\left(T_{rr}-T_{\theta \theta }\right)\right], \end{array}$$

(74) $$\begin{array}{ccc} & & \tau =0:\;T_{\theta \theta }=-B\;\frac{z}{r}; \end{array}$$

(75) $$\begin{array}{ccc} & & \frac{\partial^{\alpha }T_{zz}}{\partial \tau^{\alpha }}=a\left[\frac{\partial^{2}T_{zz}}{\partial r^{2}}+\frac{1}{r}\;\frac{\partial T_{zz}}{\partial r}-\frac{1}{r^{2}}\;T_{zz}\right], \end{array}$$

(76) $$\begin{array}{ccc} & & \tau =0:\;T_{zz}=-2\nu \;B\;\frac{z}{r}; \end{array}$$

(77) $$\begin{array}{ccc} & & \frac{\partial^{\alpha }T_{r\theta }}{\partial \tau^{\alpha }}=a\left[\frac{\partial^{2}T_{r\theta }}{\partial r^{2}}+\frac{1}{r}\;\frac{\partial T_{r\theta }}{\partial r}-\frac{5}{r^{2}}\;T_{r\theta }+\frac{2}{r^{2}}\;\left(T_{rr}-T_{\theta \theta }\right)\right], \end{array}$$

(78) $$\begin{array}{ccc} & & \tau =0:\;T_{r\theta }=B\;\frac{z}{r}; \end{array}$$

(79) $$\begin{array}{ccc} & & \frac{\partial^{\alpha }T_{rz}}{\partial \tau^{\alpha }}=a\left[\frac{\partial^{2}T_{rz}}{\partial r^{2}}+\frac{1}{r}\;\frac{\partial T_{rz}}{\partial r}-\frac{2}{r^{2}}\;\left(T_{rz}-T_{\theta z}\right)\right], \end{array}$$

(80) $$\begin{array}{ccc} & & \tau =0:\;T_{rz}=-B\left(1-2\nu \right)\ln\left(r/l\right); \end{array}$$

(81) $$\begin{array}{ccc} & & \frac{\partial^{\alpha }T_{\theta z}}{\partial \tau^{\alpha }}=a\left[\frac{\partial^{2}T_{\theta z}}{\partial r^{2}}+\frac{1}{r}\;\frac{\partial T_{\theta z}}{\partial r}+\frac{2}{r^{2}}\;\left(T_{rz}-T_{\theta z}\right)\right], \end{array}$$

(82) $$\begin{array}{ccc} & & \tau =0:\;T_{\theta z}=-B\left[\left(1-2\nu \right)\ln\left(r/l\right)+1\right]. \end{array}$$

First, we study Equations (71)–(78). To split a system of coupled equations, we introduce the new unknown functions

(83) $$\begin{array}{ccc} f & = & T_{rr}+T_{\theta \theta }, \end{array}$$

(84) $$\begin{array}{ccc} g & = & T_{rr}-T_{\theta \theta }+2T_{r\theta }, \end{array}$$

(85) $$\begin{array}{ccc} h & = & T_{rr}-T_{\theta \theta }-2T_{r\theta }, \end{array}$$

and obtain

(86) $$\begin{array}{ccc} & & \frac{\partial^{\alpha }f}{\partial \tau^{\alpha }}=a\left[\frac{\partial^{2}f}{\partial r^{2}}+\frac{1}{r}\;\frac{\partial f}{\partial r}-\frac{1}{r^{2}}\;f\right], \end{array}$$

(87) $$\begin{array}{ccc} & & \tau =0:\;f=-2B\;\frac{z}{r}; \end{array}$$

(88) $$\begin{array}{ccc} & & \frac{\partial^{\alpha }g}{\partial \tau^{\alpha }}=a\left[\frac{\partial^{2}g}{\partial r^{2}}+\frac{1}{r}\;\frac{\partial g}{\partial r}-\frac{1}{r^{2}}\;g\right], \end{array}$$

(89) $$\begin{array}{ccc} & & \tau =0:\;g=2B\;\frac{z}{r}; \end{array}$$

(90) $$\begin{array}{ccc} & & \frac{\partial^{\alpha }h}{\partial \tau^{\alpha }}=a\left[\frac{\partial^{2}h}{\partial r^{2}}+\frac{1}{r}\;\frac{\partial h}{\partial r}-\frac{9}{r^{2}}\;h\right], \end{array}$$

(91) $$\begin{array}{ccc} & & \tau =0:\;h=-2B\;\frac{z}{r}. \end{array}$$

The Laplace transform with respect to the nonlocality parameter τ and the Hankel transform with respect to the radial coordinate *r* (of the first order for the functions *f* and *g* and of the third order for the function *h*) give

(92) $$f=-2Bz\int_{0}^{\infty }E_{\alpha }\left(-a\xi^{2}\tau^{\alpha }\right)J_{1}\left(r\xi \right)\;d\xi ,$$

(93) $$g=2Bz\int_{0}^{\infty }E_{\alpha }\left(-a\xi^{2}\tau^{\alpha }\right)J_{1}\left(r\xi \right)\;d\xi ,$$

(94) $$h=-2Bz\int_{0}^{\infty }E_{\alpha }\left(-a\xi^{2}\tau^{\alpha }\right)J_{3}\left(r\xi \right)\;d\xi .$$

The components of the nonlocal stress tensor have the following form:

(95) $$t_{rr}=-\frac{Bz}{2}\int_{0}^{\infty }E_{\alpha }\left(-a\xi^{2}\tau^{\alpha }\right)\left[J_{1}\left(r\xi \right)+J_{3}\left(r\xi \right)\right]d\xi \;\sin\theta ,$$

(96) $$t_{\theta \theta }=-\frac{Bz}{2}\int_{0}^{\infty }E_{\alpha }\left(-a\xi^{2}\tau^{\alpha }\right)\left[3J_{1}\left(r\xi \right)-J_{3}\left(r\xi \right)\right]d\xi \;\sin\theta ,$$

(97) $$t_{zz}=-2B\;\nu \;z\int_{0}^{\infty }E_{\alpha }\left(-a\xi^{2}\tau^{\alpha }\right)J_{1}\left(r\xi \right)\;d\xi \;\sin\theta ,$$

(98) $$t_{r\theta }=Bz\int_{0}^{\infty }E_{\alpha }\left(-a\xi^{2}\tau^{\alpha }\right)\left[J_{1}\left(r\xi \right)+J_{3}\left(r\xi \right)\right]d\xi \;\cos\theta .$$

For α=1, using integrals (A18) and (A19) from Appendix B, we obtain

(99) $$t_{rr}=-\frac{Bz}{r}\left\{1-\frac{4a\tau }{r^{2}}\left[1-\exp\left(-\frac{r^{2}}{4a\tau }\right)\right]\right\}\sin\theta ,$$

(100) $$t_{\theta \theta }=-\frac{Bz}{r}\left\{-1+\left(2+\frac{4a\tau }{r^{2}}\right)\left[1-\exp\left(-\frac{r^{2}}{4a\tau }\right)\right]\right\}\sin\theta ,$$

(101) $$t_{zz}=-\frac{2B\;\nu \;z}{r}\left[1-\exp\left(-\frac{r^{2}}{4a\tau }\right)\right]\sin\theta ,$$

(102) $$t_{r\theta }=\frac{Bz}{r}\left\{1-\frac{4a\tau }{r^{2}}\left[1-\exp\left(-\frac{r^{2}}{4a\tau }\right)\right]\right\}\cos\theta .$$

For α→0, taking into account integrals (A15)–(A17) from Appendix B and the recurrence equations for the modified Bessel functions $K_{n}\left(r\right)$, we obtain

(103) $$t_{rr}=-\frac{Bz}{r}\left[1-\frac{4a}{r^{2}}+2K_{2}\left(\frac{r}{\sqrt{a}}\right)\right]\sin\theta ,$$

(104) $$t_{\theta \theta }=-\frac{Bz}{r}\left[1+\frac{4a}{r^{2}}-2K_{2}\left(\frac{r}{\sqrt{a}}\right)-\frac{2r}{\sqrt{a}}\;K_{1}\left(\frac{r}{\sqrt{a}}\right)\right]\sin\theta ,$$

(105) $$t_{zz}=-\frac{2B\;\nu \;z}{r}\left[1-\frac{r}{\sqrt{a}}\;K_{1}\left(\frac{r}{\sqrt{a}}\right)\right]\sin\theta ,$$

(106) $$t_{r\theta }=\frac{Bz}{r}\left[1-\frac{4a}{r^{2}}+2K_{2}\left(\frac{r}{\sqrt{a}}\right)\right]\cos\theta .$$

The solutions for the particular cases α=1 and α→0 were considered in [46].

**Figure 4:**
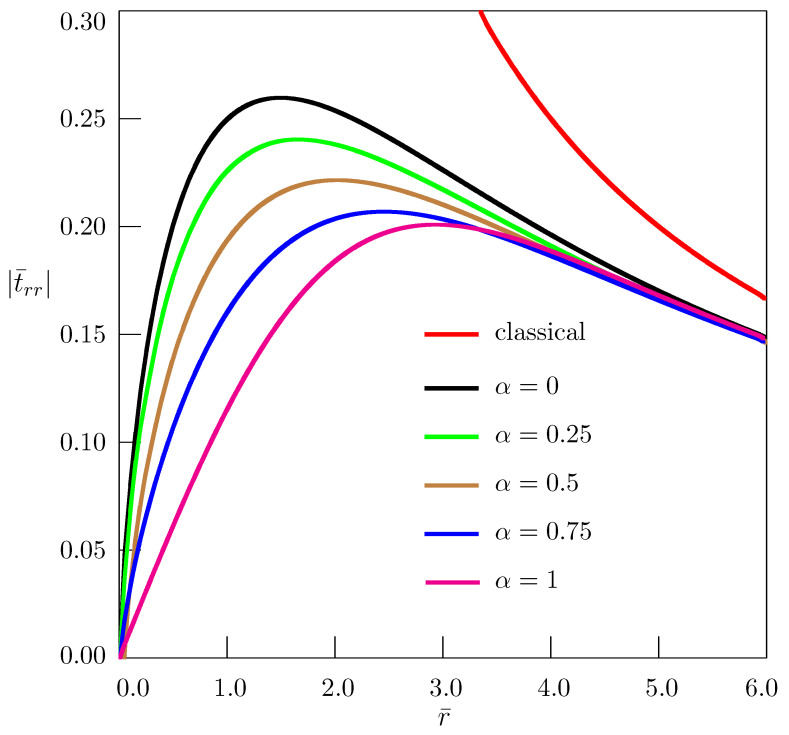
Dependence of stress ${\bar{t}}_{rr}$ on distance for straight twist disclination.

**Figure 5:**
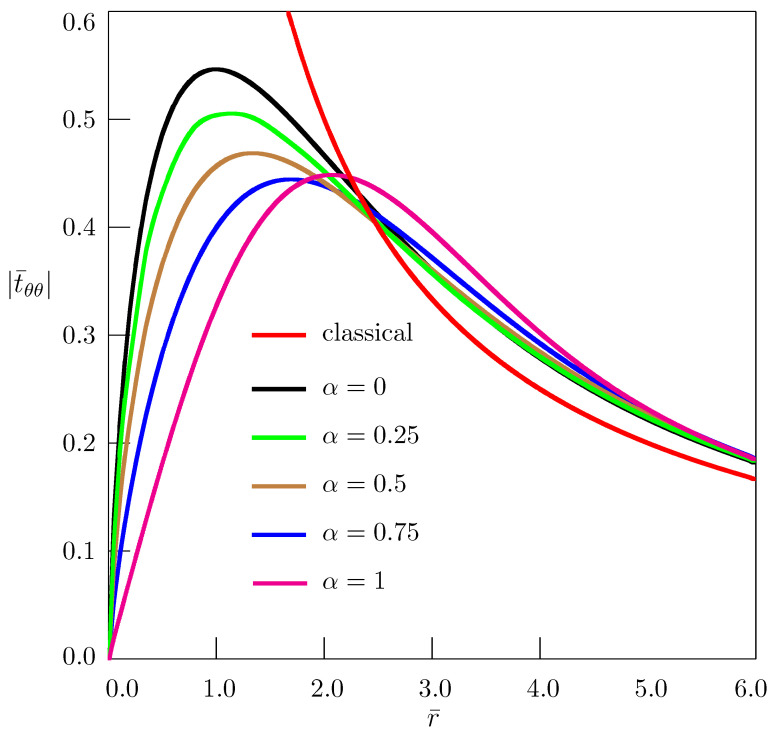
Dependence of stress ${\bar{t}}_{\theta \theta }$ on distance for straight twist disclination.

**Figure 6:**
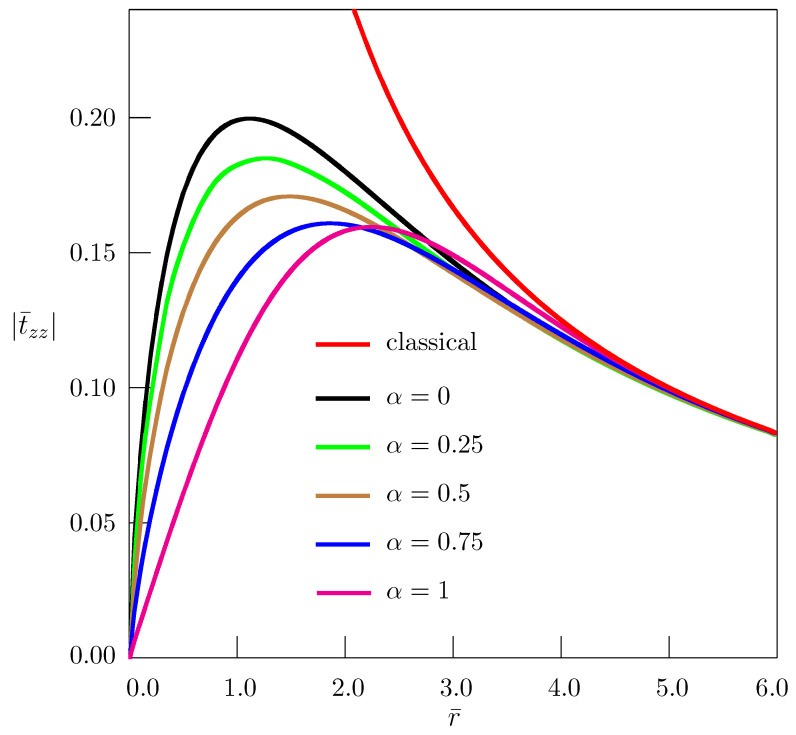
Dependence of stress ${\bar{t}}_{zz}$ on distance for straight twist disclination.

Figure 4, Figure 5 and Figure 6 present the dependence of nondimensional stresses on distance. Nondimensional quantities are introduced as

(107) $${\bar{t}}_{ij}=\frac{t_{ij}}{B},\;\bar{r}=\frac{r}{\sqrt{a}\tau^{\alpha /2}},\;\bar{z}=\frac{z}{\sqrt{a}\tau^{\alpha /2}}.$$

In our computation, we have taken ν=0.25, θ=π/2, $\bar{z}=1$.

The classical elasticity solution (red curves in Figure 4, Figure 5 and Figure 6) has nonphysical singularity at the disclination line. The solutions obtained in the framework of the fractional nonlocal elasticity are free from such singularities. Depending on the order of the fractional derivative, the approximate estimate of maximum values of stresses, based on the numerical simulation and using Equations (64) and (107) for $\Omega_{2}=\pi /30$, gives quite acceptable values: $t_{rr}\approx 0.0044\;\mu -0.0058\;\mu$, $t_{\theta \theta }\approx 0.0098\;\mu -0.0121\;\mu$, $t_{zz}\approx 0.0035\;\mu -0.0044\;\mu$.

Next, we deal with Equations (79)–(82) and introduce two new unknown functions

(108) $$\begin{array}{ccc} p & = & T_{rz}+T_{\theta z}, \end{array}$$

(109) $$\begin{array}{ccc} q & = & T_{rz}-T_{\theta z}, \end{array}$$

satisfying the split system of equations

(110) $$\begin{array}{ccc} & & \frac{\partial^{\alpha }p}{\partial \tau^{\alpha }}=a\left(\frac{\partial^{2}p}{\partial r^{2}}+\frac{1}{r}\;\frac{\partial p}{\partial r}\right), \end{array}$$

(111) $$\begin{array}{ccc} & & \tau =0:\;p=-B\left[2\left(1-2\nu \right)\ln\left(r/l\right)+1\right], \end{array}$$

(112) $$\begin{array}{ccc} & & \frac{\partial^{\alpha }q}{\partial \tau^{\alpha }}=a\left(\frac{\partial^{2}q}{\partial r^{2}}+\frac{1}{r}\;\frac{\partial q}{\partial r}-\frac{4}{r^{2}}\;q\right), \end{array}$$

(113) $$\begin{array}{ccc} & & \tau =0:\;q=B. \end{array}$$

A system of equations of such a type was considered in the case of the wedge disclination (see the corresponding Equations (27)–(30)). Repeating the examination carried out in Section 2, we can immediately write the sought components of the nonlocal stress tensor (compare Equations (49), (50), (114) and (115)):

(114) $$\begin{array}{ccc} t_{rz}\left(r,\tau \right) & = & \{-\frac{1}{2}\;B\left(1-2\nu \right)\left[2\ln\left(\frac{r}{\sqrt{a}\tau^{\alpha /2}}\right)+\frac{1}{1-2\nu }-\alpha \gamma \right]\\ & - & B\left(1-2\nu \right)\int_{0}^{\infty }\frac{1-E_{\alpha }\left(-a\xi^{2}\tau^{\alpha }\right)}{\xi }\;J_{0}\left(r\xi \right)d\xi \\ & + & B\int_{0}^{\infty }\frac{E_{\alpha }\left(-a\xi^{2}\tau^{\alpha }\right)}{\xi }\;J_{2}\left(r\xi \right)d\xi \;\}\sin\theta , \end{array}$$

(115) $$\begin{array}{ccc} t_{\theta z}\left(r,\tau \right) & = & \{-\frac{1}{2}\;B\left(1-2\nu \right)\left[2\ln\left(\frac{r}{\sqrt{a}\tau^{\alpha /2}}\right)+\frac{1}{1-2\nu }-\alpha \gamma \right]\\ & - & B\left(1-2\nu \right)\int_{0}^{\infty }\frac{1-E_{\alpha }\left(-a\xi^{2}\tau^{\alpha }\right)}{\xi }\;J_{0}\left(r\xi \right)d\xi \\ & - & B\int_{0}^{\infty }\frac{E_{\alpha }\left(-a\xi^{2}\tau^{\alpha }\right)}{\xi }\;J_{2}\left(r\xi \right)d\xi \;\}\cos\theta . \end{array}$$

Similarly to Equations (53)–(56), the solutions for the limiting cases α=1 and α→0 can also be written substituting the components $t_{rr}$ and $t_{\theta \theta }$ by the components $t_{rz}$ and $t_{\theta z}$, respectively.

## 5. Concluding Remarks

We have studied the nonlocal stresses caused by straight wedge and twist disclinations in an infinite medium in the framework of the new theory of fractional nonlocal elasticity. In this new theory, the constitutive equation for the nonlocal stress tensor is formulated as an integral of the local stress tensor with the nonlocality kernel (the weight function) being the Green function of the Cauchy problem for the fractional diffusion operator with the Caputo fractional derivative of the order 0<α<1 with respect to the nonlocality parameter. The existence of different nonlocality kernels allows one to choose the most suitable one for the specific problem. The particular cases of the solutions for α→1 and α→0 coincide with those known in the literature. The obtained stress fields do not contain the nonphysical singularities at the disclination line that appear in the classical local solutions. The approximate estimates of maximum values of nonlocal stress are quite acceptable from a physical point of view and are comparable with similar estimates for dislocations carried out by Eringen [40,41,42,43,44] (see also [73,74,75]). The stress problems for straight disclinations are more complicated than the corresponding problems for straight dislocations due to the logarithmic terms in the local elasticity solutions. The nonlocal theory eliminates stress singularities at the origin, but the logarithmic divergence remains at infinity. Such a divergence at infinity disappears in a finite domain, for example, for loading free boundary conditions (see, for example, [21,46]). The proposed fractional nonlocal theory of elasticity can be useful for better matching the theory of elasticity and atomic lattice theory. The importance of such matching was emphasized in many publications.

In the description of defects in solids, theories that use couple stress are of great importance. In future studies, we will analyze this aspect of nonlocal elasticity theory. The first steps in this direction have been made in the publications [76,77].

## Data Availability

The original contributions presented in this study are included in the article. Further inquiries can be directed to the corresponding author.

## Nomenclature

The following abbreviations are used in this manuscript:

*A*	proportionality coefficient for wedge disclination stresses
*a*	coefficient in the diffusion equation for the nonlocal kernel
*B*	proportionality coefficient for twist disclination stresses
$E_{\alpha }$	Mittag–Leffler function
$E_{\alpha }$	exponential integral
e	linear strain tensor
*f*	auxiliary function
*g*	auxiliary function
*h*	auxiliary function
I	unit tensor
$I_{n}$	modified Bessel function
$J_{n}$	Bessel function
*K*	nonlocality kernel
$K_{n}$	modified Bessel function
*l*	characteristic length
*p*	auxiliary function
*q*	auxiliary function
*r*	cylindrical coordinate
*s*	Laplace transform variable
$T_{ij}$	coefficients of expressions for components of nonlocal stress tensor $t_{ij}$
t	nonlocal stress tensor
x	reference point
$x^{\prime }$	running point
*z*	cylindrical coordinate
α	order of fractional derivative with respect to nonlocality parameter
Γ	gamma function
γ	Euler constant
Δ	Laplace operator
δ	Dirac’s delta function
θ	cylindrical coordinate
λ	Lamé constant
μ	Lamé constant
ν	Poisson ratio
ξ	Hankel transform variable
σ	local stress tensor
τ	nonlocality parameter
Ω	Frank vector

## Appendix A

The components of the Laplacian of the symmetric second-order tensor in cylindrical coordinates $\left(r,\theta ,z\right)$ were obtained in [46] (see also [44,78,79]):

(A1) $${\left(\Delta t\right)}_{rr}=\Delta \left(t_{rr}\right)-\frac{4}{r^{2}}\;\frac{\partial t_{r\theta }}{\partial \theta }-\frac{2}{r^{2}}\;\left(t_{rr}-t_{\theta \theta }\right),$$

(A2) $${\left(\Delta t\right)}_{\theta \theta }=\Delta \left(t_{\theta \theta }\right)+\frac{4}{r^{2}}\;\frac{\partial t_{r\theta }}{\partial \theta }+\frac{2}{r^{2}}\;\left(t_{rr}-t_{\theta \theta }\right),$$

(A3) $${\left(\Delta t\right)}_{r\theta }=\Delta \left(t_{r\theta }\right)-\frac{4}{r^{2}}\;t_{r\theta }+\frac{2}{r^{2}}\;\frac{\partial }{\partial \theta }\left(t_{rr}-t_{\theta \theta }\right),$$

(A4) $${\left(\Delta t\right)}_{rz}=\Delta \left(t_{rz}\right)-\frac{1}{r^{2}}\;t_{rz}-\frac{2}{r^{2}}\;\frac{\partial t_{\theta z}}{\partial \theta },$$

(A5) $${\left(\Delta t\right)}_{\theta z}=\Delta \left(t_{\theta z}\right)-\frac{1}{r^{2}}\;t_{\theta z}+\frac{2}{r^{2}}\;\frac{\partial t_{rz}}{\partial \theta },$$

(A6) $${\left(\Delta t\right)}_{zz}=\Delta \left(t_{zz}\right),$$

where

(A7) $$\Delta f=\frac{\partial^{2}f}{\partial r^{2}}+\frac{1}{r}\;\frac{\partial f}{\partial r}+\frac{1}{r^{2}}\;\frac{\partial^{2}f}{\partial \theta^{2}}+\frac{\partial^{2}f}{\partial z^{2}}.$$

## Appendix B

In this Appendix, we present the integrals used in the paper.

Integrals (A8)–(A10) are taken from [80]:

(A8) $$\int_{0}^{\infty }K_{0}\left(ar\right)J_{0}\left(br\right)r\;dr=\frac{1}{a^{2}+b^{2}},$$

(A9) $$\int_{0}^{\infty }K_{2}\left(ar\right)J_{2}\left(br\right)r\;dr=\frac{b^{2}}{a^{2}\left(a^{2}+b^{2}\right)},$$

(A10) $$\int_{0}^{\infty }J_{2}\left(bx\right)x\;dx=\frac{2}{b^{2}}.$$

Integrals (A11) and (A12) are evaluated by the authors:

(A11) $$\int_{0}^{\infty }\frac{1}{x}\left(1-e^{-ax^{2}}\right)J_{0}\left(bx\right)dx=\frac{1}{2}\;E_{1}\left(\frac{b^{2}}{4a}\right),$$

(A12) $$\int_{0}^{\infty }\frac{1}{x^{2}}\left(1-e^{-ax^{2}}\right)J_{1}\left(bx\right)dx=\frac{a}{b}\left[1-E_{2}\left(\frac{b^{2}}{4a}\right)\right].$$

Here, $E_{n}\left(z\right)$ is the exponential integral [71]

(A13) $$E_{n}\left(z\right)=\int_{1}^{\infty }\frac{e^{-zt}}{t^{n}}\;dt$$

with the following recurrence equation:

(A14) $$E_{n+1}\left(z\right)=\frac{1}{n}\left[e^{-z}-z\;E_{n}\left(z\right)\right].$$

For the exponential integral $E_{n}\left(z\right)$, we have used the notation $E_{n}\left(z\right)$ to prevent confusion with the Mittag–Leffler function $E_{\alpha }\left(z\right)$.

(A15) $$\int_{0}^{\infty }\frac{x}{x^{2}+a^{2}}\;J_{0}\left(bx\right)dx=K_{0}\left(ab\right),$$

(A16) $$\int_{0}^{\infty }\frac{1}{x^{2}+a^{2}}\;J_{1}\left(bx\right)dx=\frac{1}{a}\left[\frac{1}{ab}-K_{1}\left(ab\right)\right],$$

(A17) $$\int_{0}^{\infty }\frac{1}{x^{2}+a^{2}}\;J_{3}\left(bx\right)dx=\frac{1}{a}\left[\frac{1}{ab}-\frac{8}{a^{3}b^{3}}+K_{3}\left(ab\right)\right],$$

(A18) $$\int_{0}^{\infty }\exp\left(-ax^{2}\right)\;J_{1}\left(bx\right)dx=\frac{1}{b}\left[1-\exp\left(-\frac{b^{2}}{4a}\right)\right],$$

(A19) $$\int_{0}^{\infty }\exp\left(-ax^{2}\right)\;J_{3}\left(bx\right)dx=\frac{1}{b}\left[1-\frac{8a}{b^{2}}+\left(1+\frac{8a}{b^{2}}\right)\exp\left(-\frac{b^{2}}{4a}\right)\right].$$

$K_{n}\left(z\right)$ is the modified Bessel function of the second kind.
